# Neural Measures of Pitch Processing in EEG Responses to Running Speech

**DOI:** 10.3389/fnins.2021.738408

**Published:** 2021-12-21

**Authors:** Florine L. Bachmann, Ewen N. MacDonald, Jens Hjortkjær

**Affiliations:** ^1^Hearing Systems Section, Department of Health Technology, Technical University of Denmark, Lyngby, Denmark; ^2^Department of Systems Design Engineering, University of Waterloo, Waterloo, ON, Canada; ^3^Danish Research Centre for Magnetic Resonance, Centre for Functional and Diagnostic Imaging and Research, Copenhagen University Hospital - Amager and Hvidovre, Copenhagen, Denmark

**Keywords:** neural tracking, subcortical, running speech, auditory brainstem response, temporal response function, encoding model, EEG

## Abstract

Linearized encoding models are increasingly employed to model cortical responses to running speech. Recent extensions to subcortical responses suggest clinical perspectives, potentially complementing auditory brainstem responses (ABRs) or frequency-following responses (FFRs) that are current clinical standards. However, while it is well-known that the auditory brainstem responds both to transient amplitude variations and the stimulus periodicity that gives rise to pitch, these features co-vary in running speech. Here, we discuss challenges in disentangling the features that drive the subcortical response to running speech. Cortical and subcortical electroencephalographic (EEG) responses to running speech from 19 normal-hearing listeners (12 female) were analyzed. Using forward regression models, we confirm that responses to the rectified broadband speech signal yield temporal response functions consistent with wave V of the ABR, as shown in previous work. Peak latency and amplitude of the speech-evoked brainstem response were correlated with standard click-evoked ABRs recorded at the vertex electrode (Cz). Similar responses could be obtained using the fundamental frequency (F0) of the speech signal as model predictor. However, simulations indicated that dissociating responses to temporal fine structure at the F0 from broadband amplitude variations is not possible given the high co-variance of the features and the poor signal-to-noise ratio (SNR) of subcortical EEG responses. In cortex, both simulations and data replicated previous findings indicating that envelope tracking on frontal electrodes can be dissociated from responses to slow variations in F0 (relative pitch). Yet, no association between subcortical F0-tracking and cortical responses to relative pitch could be detected. These results indicate that while subcortical speech responses are comparable to click-evoked ABRs, dissociating pitch-related processing in the auditory brainstem may be challenging with natural speech stimuli.

## 1. Introduction

Subcortical responses to sound measured with electroencephalography (EEG) have traditionally relied on evoked responses to short stimuli averaged over thousands of repetitions. Numerous studies have more recently used linear stimulus-response models to quantify neural tracking of running natural speech or other naturalistic stimuli in the cortex (Lalor et al., 2009; Ding and Simon, 2012; Di Liberto et al., 2020; Kulasingham et al., 2020; Kurthen et al., 2021). These efforts have now been extended to the subcortical auditory system (Forte et al., 2017; Maddox and Lee, 2018; Etard et al., 2019; Polonenko and Maddox, 2021; Van Canneyt et al., 2021a,b,c), leveraging the fact that EEG responses to fast acoustic variations are dominated by subcortical sources (Bidelman, 2018; Saiz-Alía and Reichenbach, 2020). The idea of using deconvolution to model brainstem EEG responses has previously been proposed in the context of evoked responses, i.e., stimulus-triggered averages with multiple repetitions of short sounds (Elberling, 1978; Dau, 2003). Goldstein and Kiang (1958) introduced the concept that the measured electrode response in the far-field can be understood as the convolution of a unit waveform with the underlying neural population activity. To estimate a “unitary response” function at the brainstem level, Dau (2003) deconvolved measured click-evoked ABRs at 60 dB SPL (sound pressure level) with simulated auditory nerve activity obtained from a computational auditory nerve model. The unitary response function was then used to model both ABRs and FFRs for different stimulus and level conditions beyond those used to estimate the response function. Lalor et al. (2009) later suggested that response functions can similarly be estimated for running stimuli like speech, by deconvolving unaveraged electrode responses with the amplitude envelope of the continuous stimulus (Lalor and Foxe, 2010).

The ability to measure subcortical responses to running speech has a number of appealing perspectives in auditory neuroscience as well as in clinical audiology. In contrast to listening to repeating and thus highly predictable short sounds, listening to running speech is a relevant task in daily life. It enables more naturalistic listening experiments where results are potentially more transferable to real-life situations (Hamilton and Huth, 2020). Using speech, cognitive top-down processes that may not play a role for short isolated syllables can potentially be addressed. It yields possibilities for investigating subcortical effects of language learning, differences between languages, or assessing responses to pitch contours in their semantic context (Llanos et al., 2021). Measuring distinct subcortical responses to different speech features would not only help to shed light on the nature of neural auditory information processing mechanisms in the midbrain, but might also offer new perspectives for clinical intervention. For example, perceptual weighting of envelope and pitch cues for perceiving lexical tones may change with hearing impairment (Wang et al., 2011), and distinct neural readouts may support tailoring hearing solutions to listeners' needs. Furthermore, simultaneous EEG measures of both subcortical and cortical responses to the same naturalistic speech stimulus can potentially be used to investigate interactions along the auditory pathway. This way, speech processing from fundamental to higher-order aspects can be studied with the same data (Brodbeck and Simon, 2020). Changes in the interaction between peripheral and central auditory processing may be particularly relevant in the study of aging (Bidelman et al., 2014) and hearing loss (Presacco et al., 2019).

However, running speech also comes with the challenge of dissociating the features driving the measured neural response (Hamilton and Huth, 2020). Many relevant features co-vary in natural speech, challenging the interpretation of stimulus-response models. Previous studies of subcortical EEG responses to running speech have focused on different acoustic features of the speech signal. Maddox and Lee (2018) used the half-wave rectified broadband speech signal to predict the running subcortical EEG via linear regression. They showed a high degree of consistency between speech-derived response functions and conventional click-evoked ABRs. In particular, speech-ABRs showed a prominent peak at latencies corresponding to wave V of conventional click-ABRs (6.17 ± 0.31 ms). Polonenko and Maddox (2021) further showed that when the glottal pulse train is used for response estimation, speech resynthesized to have sharp peaks in the pressure waveform additionally yielded earlier wave-I-like components in the speech-ABR. Forte et al. (2017), on the other hand, examined subcortical responses to the F0 of running speech signals. To model the stimulus-response relation, they computed the cross-correlation between both a periodicity feature (an extracted F0 waveform) as well as its Hilbert transform and the EEG. These were treated as the real and imaginary parts of a complex cross-correlation function, and the magnitude was interpreted as the neural response. Peak latencies occurred around 6–10 ms, corresponding to latencies observed with short periodic stimuli like speech syllables or tones (Skoe and Kraus, 2010). In a later study, Etard et al. (2019) instead simply applied a band-pass filter around the F0 of the speech signal and obtained similar results as Forte et al. (2017). Van Canneyt et al. (2021c) similarly used F0 band-passed speech and regularized linear regression (rather than cross-correlation) to predict the running EEG signal. They found different early response peak latencies for their four female- (12.29, 10.24, 10.24, 7.17 ms) and two male-narrated (13.31, 14.34 ms) stories (Van Canneyt et al., 2021c), suggesting an influence of F0 on response latency. Thus, the studies of Forte et al. (2017) or Etard et al. (2019) focusing on subcortical pitch-related processing reported response peak latencies comparable to the speech-ABR studies of Maddox and Lee (2018) or Polonenko and Maddox (2021). However, the responses also showed qualitative differences. While Maddox and Lee (2018) obtained an ABR-like waveform morphologically similar to click-ABRs, Forte et al. (2017), Etard et al. (2019), and also Van Canneyt et al. (2021c) showed F0-responses with broader response peaks at later latencies.

Together, these results indicate that modeled measures of speech tracking in the auditory brainstem are consistent with known evoked response measures. However, the approaches differ both in terms of the considered speech features and the applied stimulus-response analysis. It therefore remains unclear whether observed differences in response waveforms stem from these methodological decisions highlighting different parts of the same underlying response, or indicate that distinct responses to pitch can be extracted. It is commonly accepted that the auditory brainstem responds to both the temporal fine structure of periodic stimuli (as reflected in FFRs) and to broadband amplitude variations in transient stimuli (as reflected in click-ABRs). Yet, these features are highly correlated in natural speech and might not be dissociable given the relatively low SNR of subcortical EEG measurements.

While the exact pitch processing mechanisms along the central auditory pathway remain debated, neuronal firing intervals matching the fundamental period of periodic sounds has been proposed as a temporal representation of pitch in the auditory nerve and brainstem (Hewitt and Meddis, 1992; Cariani and Delgutte, 1996; see e.g., Oxenham, 2013 for an overview). To probe pitch-related processing in the auditory brainstem, FFRs to periodic stimuli are often used (Krishnan et al., 2010; Bidelman and Krishnan, 2011; Krishnan and Gandour, 2017). The FFR elicited by harmonic sounds is argued to be predictive of speech-in-noise performance (Anderson et al., 2011, 2013), and speech understanding in reverberation (Fujihira and Shiraishi, 2015). Tonal language speakers show stronger FFR responses to lexically relevant changes in the F0 track (Krishnan et al., 2005; Krishnan and Gandour, 2017). Phase-locked activity in the brainstem to the F0 has been observed for missing-fundamental stimuli, i.e., stimuli that elicit a pitch percept despite the absence of energy at the F0 (Smith et al., 1978; Galbraith, 1994). This might indicate that F0-tracking at the level of the brainstem reflects a pitch-extraction mechanism. It remains unclear, however, whether pitch processing in the brainstem can be investigated with running speech where pitch co-varies with other acoustic features.

In the cortex, recent studies have also investigated pitch tracking with running speech (Tang et al., 2017; Teoh et al., 2019; Li et al., 2021; Llanos et al., 2021). In a recent electrocorticography study, Tang et al. (2017) showed cortical tracking of relative pitch contours by high-gamma band activity. The cortical tracking of the relative changes in slowly varying F0 contours (rather than its temporal fine structure) was also shown by Teoh et al. (2019) using low-frequency EEG responses to running speech. To dissociate pitch and envelope processing, Teoh et al. (2019) used model comparisons and showed that adding relative pitch (a normalized F0 trajectory) to a regression model of the low-passed envelope improved prediction of the running speech EEG. Tracking of the relative pitch was absent for noise-vocoded stimuli. Yet, it remains unclear whether a similar dissociation of responses to the temporal fine structure of F0 can be achieved in the brainstem.

In this study, we compared neural responses to different pitch-related features of running speech with the aim to shed light on the current ambiguities. Specifically, we compared models of subcortical responses to running speech with either F0 periodicity or with the broadband waveform of the speech signal. We also examined the degree to which cortical responses to slowly varying pitch contours of the speech signal can be dissociated from cortical envelope tracking as reported in recent work.

## 2. Methods

### 2.1. Data Acquisition

Participants listened to an audio book and click trains while their neural activity was recorded with an EEG system. Data from 20 (13 female) young native Danish speakers without any history of psychiatric or neurological diseases were recorded. Participants were required to have pure-tone thresholds better than 25 dB hearing level in both ears (measured at standard audiometric frequencies: 250 Hz, 500 Hz, 1 kHz, 2 kHz, 4 kHz, and 8 kHz), and professional musicians were excluded. The data from one participant that did not match these criteria was excluded from further analysis, after which the participant sample consisted of 19 (12 female) people (*M*_*age*_ = 23.13 ± 2.30). All henceforth reported statistics focus on the included participants. Each participant provided written informed consent, and all experiments were approved by the Science-Ethics Committee for the Capital Region of Denmark (reference H-16036391).

Measurements were conducted in a soundproof, electrically shielded listening booth. Participants were seated in a comfortable chair in front of a computer screen. Experiment presentation and data acquisition were controlled from outside the booth. The audio book was presented at 65 dB SPL through ER-2 insert earphones (Etymotic Research), with a sampling frequency of 44.1 kHz. Delay from trigger to transducer activity was 0.18 ms, and the distance from the transducers to the ear drums caused an additional presentation delay of 0.87 ms. The total delay of 1.05 ms was accounted for in the analysis. The beginning of the Danish version of *Lord of the Flies* by William Golding, read by a male narrator with a F0 of around 107 Hz, was used as audio book. Longer pauses in the audio book recording were restricted to 450 ms, and the recording was cut into trial segments of 50 s duration. The experiment consisted of 36 trials. To ensure that participants attended the story, three multiple-choice questions were asked after every trial. For each segment, one of the three comprehension questions was presented to the participant prior to listening to the segment. Accuracy on these control questions was above 80% for all included participants (*M*_*correct*_ = 91.13% ± 4.28%). To familiarize themselves with the experimental procedure, participants completed a short training session consisting of two trials before starting the experiment. Data from the training session were not included in the analysis. The experiment was implemented using the Psychtoolbox (Kleiner et al., 2007) in Matlab (The MathWorks Inc., 2015).

To compare speech EEG recordings with standard ABRs (e.g., Maddox and Lee, 2018), click-ABR responses were obtained after the speech experiment. A 10 Hz click train with alternating polarities was presented at 93 dB peak-to-peak equivalent SPL for 5 min, resulting in 3'000 click repetitions. A rectangular click shape with a duration of 80 μs (Garret and Verhulst, 2019) was used, and no jitter was applied to the click train.

The EEG was recorded using the Active Two system (BioSemi) with a sampling rate of 16'384 Hz. Electrical potentials were measured from 32 scalp electrodes placed according to the 10–20 system, and 4 external electrodes placed on the left and right mastoid bones, as well as and over and below the right eye to measure the electrooculogram (EOG).

### 2.2. Speech Feature Extraction and EEG Preprocessing

**Figure 1:**
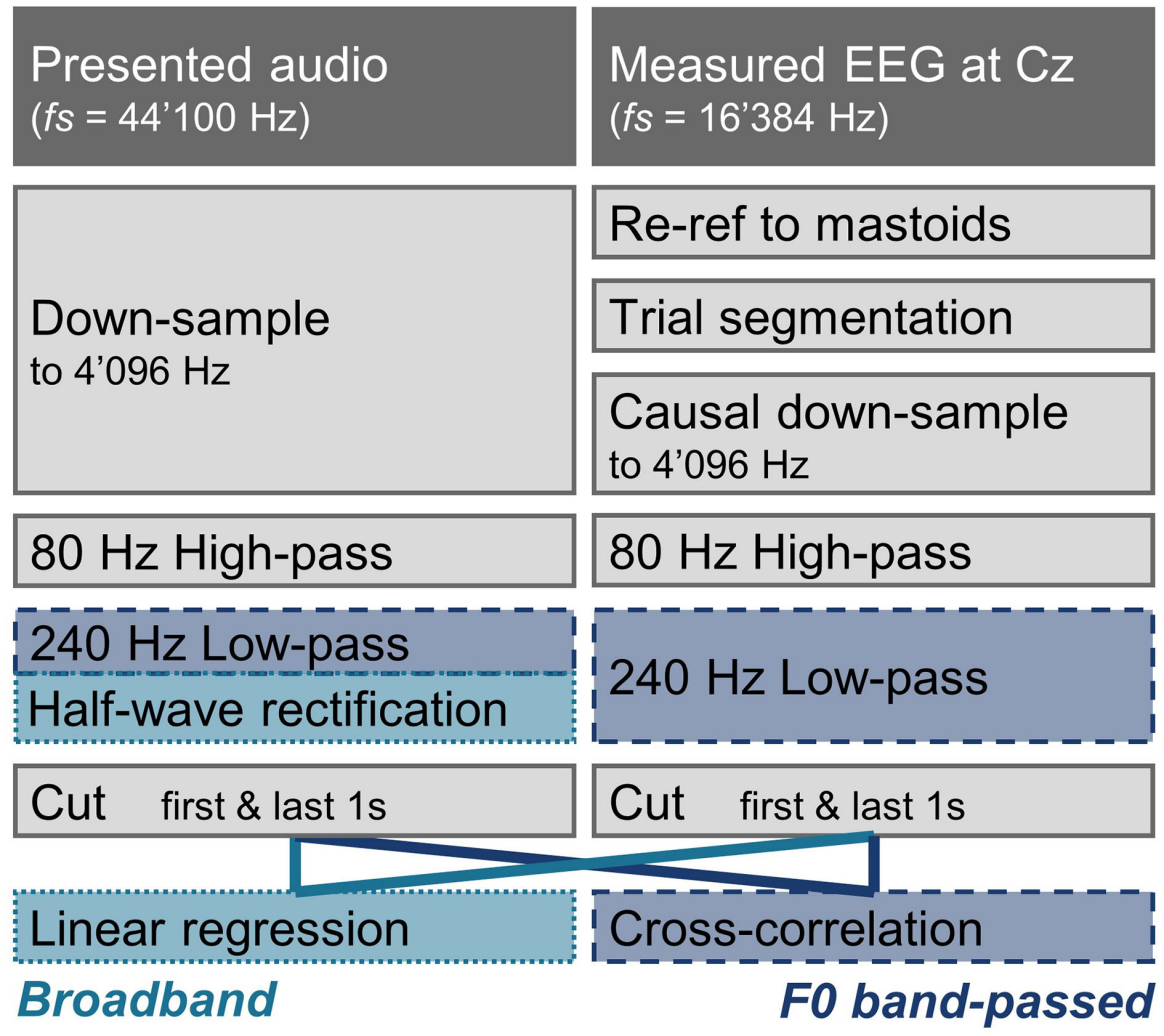
Processing pipeline of the audio and EEG in the subcortical speech response analysis. Processing steps that are identical for the broadband and the F0 band-passed features are shown in gray. Processing steps only applied for the broadband or the F0 band-passed approach are indicated with light blue (dotted line), and darker blue (dashed line), respectively. Apart from the different stimulus-response models, the two approaches mainly differ in that a half-wave rectification is applied for the broadband approach, and a 240 Hz low-pass filter is applied for the F0 band-passed approach.

In general, EEG and audio signals were preprocessed with equivalent filters whenever possible (see Figure 1), to avoid introducing differences potentially affecting the analysis. Processing was done in Matlab (The MathWorks Inc., 2020) using the FieldTrip Toolbox (Oostenveld et al., 2011). The data was first visually inspected, and electrode channels showing extreme activity indicating artifacts were excluded from the analysis (*M*_*excl*_ = 0.84 ± 1.11 channels).

#### 2.2.1. Subcortical Responses

As later and larger cortical components could have an impact on early responses, only causal filters were used on the EEG data (Maddox and Lee, 2018). The EEG was first re-referenced to the mastoid channels, and the data were segmented according the experiment trials. For one participant with noisy mastoid channel recordings, EEG was instead re-referenced to the close-by cap electrodes (T7 & T8). Both the audio and the EEG were then down-sampled to 4'096 Hz by first applying a causal anti-aliasing filter at 1'638 Hz (audio: one-pass zero-phase hamming-windowed sinc FIR corrected for filter delay, order 356, transition width 409.6 Hz; EEG: with the exception of a filter order of 132 to account for the difference in original sampling rate, the same filter design parameters were used). The EEG signal was then down-sampled by a factor of 4 (to 4'096 Hz) by taking every 4th sample. For one participant wrongly recorded at a sampling rate of 2'048 instead of 16'384 Hz, data was up-sampled using Matlab's *resample* function (anti-aliasing filter order of 34, otherwise same filter design parameters as described above). The audio was re-sampled to 4'096 Hz using the *resample* function. After down-sampling, a high-pass filter of 80 Hz was applied to both the audio and the EEG data (one-pass zero-phase hamming-windowed sinc FIR controlled for filter delay, order 676, transition width 20 Hz) to limit cortical contributions (Bidelman, 2018). The audio and EEG were further processed in two separate ways to analyse responses to F0 or to the broadband amplitude envelope. For the F0 filter, the audio and EEG were filtered with a 240 Hz low-pass filter (one-pass zero-phase hamming-windowed sinc FIR controlled for filter delay, order 226, transition width 60 Hz). For the broadband response, the audio was instead half-wave rectified and no further filtering was applied. To discard possible filtering artifacts, the first and last seconds were cut from all processed signals.

The click-ABR data were pre-processed similarly to the speech data. After the exclusion of bad electrode channels, the data were re-referenced to the mastoid electrodes, cut into trials, and down-sampled as was done for the speech data. However, before applying the 80 Hz high-pass filter, a line noise filter was applied (discrete fourier transform filter at 50, 100, and 150 Hz with bandwidths of 1, 2, and 3 Hz, respectively). Then, trials with voltages exceeding 20 μV were interpreted as including artifacts and excluded (*M*_*excl*_ = 1.87 ± 6.14% of trials). Before averaging the ABR, every trial was divided by its variance.

#### 2.2.2. Cortical Responses

For the cortical analyses, the EEG data were re-referenced to the mastoids, before re-sampling to 64 Hz (anti-aliasing filter at 30 Hz: one-pass zero-phase hamming-windowed sinc FIR controlled for filter delay, order 7210 (902 for the participant recorded at lower sampling rate), transition width 7.5 Hz). The EEG data was then high-passed at 0.5 Hz (one-pass zero-phase hamming-windowed sinc FIR controlled for filter delay, order 212, transition width 1 Hz) before trial segmentation and using the EOG electrodes for eye movement removal with joint decorrelation (de Cheveigné and Parra, 2014) following Wong et al. (2018). Finally, the EEG data were band-pass filtered between 1 and 9 Hz (high-pass filter: one-pass zerophase, order 106, transition width 2 Hz; low-pass filter: one-pass zero-phase, order 94, transition width 2.2 Hz).

**Figure 2:**
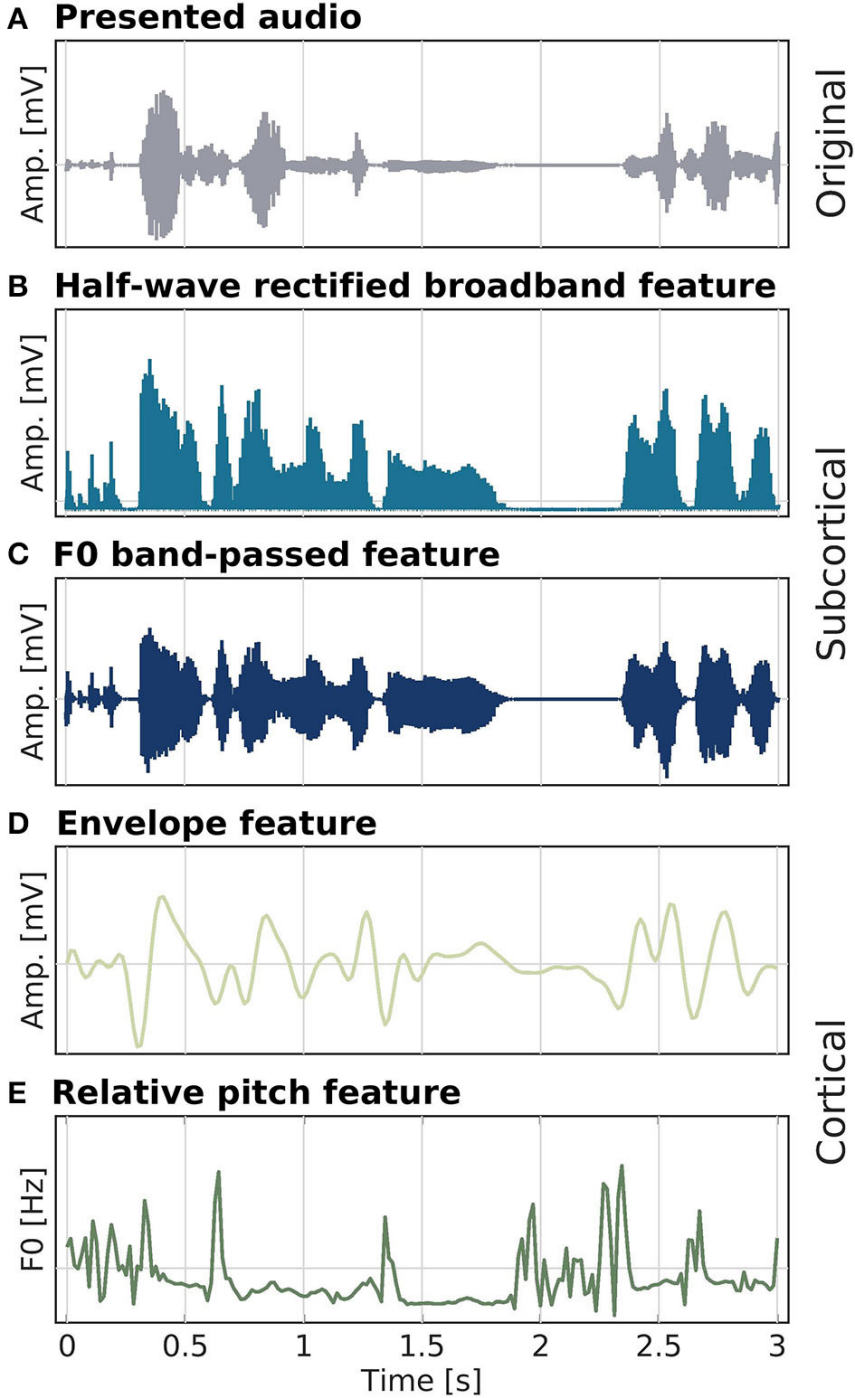
Speech stimulus **(A)** and audio features **(B–E)** used as model predictors in the different analyses. Half-wave rectified high-passed (80 Hz) broadband **(B)** and band-passed from 80 to 240 Hz, around F0 **(C)**, features were used for modeling subcortical activity. Low-frequency changes in speech amplitude **(D)** and the relative F0 trajectory **(E)** for modeling cortical activity. Amplitude and F0 values were z-standardized prior to plotting.

For the audio, we compared responses to the low-pass filtered speech envelope and to relative pitch (Teoh et al., 2019). The relative pitch feature is the relative F0 trajectory. The YIN algorithm (Cheveigné and Kawahara, 2002) was used to compute F0 estimates at every sample (limited to frequencies ranging between 80 and 240 Hz), after which z-scoring was applied. The envelope speech feature was extracted in a manner similar to Fuglsang et al. (2020). The presented audio signal was first re-sampled to 12 kHz (anti-aliasing filter at 6 kHz, two-pass zero-phase, order 98), before a gamma-tone filterbank was applied to extract 24 filter bands from 100 Hz to 4 kHz. The outputs of the filterbank were then rectified and compressed by a factor of 0.3, before averaging over the bands. The resulting signal was further down-sampled in two steps, first to 512 Hz (anti-aliasing filter at 256 Hz, two-pass zero-phase, order 620), and second to 64 Hz (anti-aliasing filter at 30 Hz, two-pass zero-phase, order 226). As a last step, the signal was band-pass filtered between 1 and 9 Hz (high-pass filter: two-pass zero-phase, order 106; low-pass filter: two-pass zero-phase, order 94). All speech features and processed EEG were z-scored at the trial level before entering into the analysis. An overview over all speech features is depicted in Figure 2.

### 2.3. Analysis

Speech responses were analyzed using forward encoding models that map between the auditory stimulus features and the EEG response. Unless otherwise stated, the models were estimated using linear ridge regressions (Tikhonov and Arsenin, 1977), or special cases thereof. With a ridge regression, the regression weights β are estimated as:


(1) $$\begin{array}{r} \hat{\beta }={\left(X^{\prime }X+\lambda I\right)}^{-1}\left(X^{\prime }Y\right) \end{array}$$

where *X* is the time-lagged stimulus feature, λ is a regularization parameter, and *Y* is the neural response data for a given EEG channel. The regression weights β yield a temporal response function (TRF) that can be interpreted as the stimulus-evoked impulse response from a neural population (Lalor et al., 2009; Ding and Simon, 2012; Crosse et al., 2021). When fitting the regularization parameter in the cortical response analysis, a 3-way nested cross-validation procedure was used in which the data were split into training, validation and test sets as described in Fuglsang et al. (2020). The λ parameter was fit to yield optimal correlation on the training (25.92 ± 0.63 trials) and validation (6.48 ± 0.50 trials) set, a procedure which was repeated 5 times before choosing the optimal λ value. Then, the prediction accuracy was computed on the independent test set (3.60 ± 0.49 trials). This procedure was repeated 10 times, and the estimated regression weights were averaged. The cortical ridge regression model was computed over lags from –312 to 812 ms, and λ was fitted for lags spanning from 47 to 266 ms.

For the subcortical response analyses, we followed previous studies to facilitate comparison. Following Maddox and Lee (2018), the EEG was regressed onto the broadband rectified audio without regularization, i.e., setting λ to 0. Without the need to fit the regularization parameter, the TRF prediction accuracy was simply evaluated using leave-one-trial-out cross-validation. The TRF was estimated for lags from –5.13 to 25.15 ms.

Responses to the F0 band-passed feature were estimated using cross-correlation following e.g., Forte et al. (2017). In a regression framework this corresponds to high regularization, whereby the relative influence of the auto-correlation term *X*′*X* in Equation (1) is minimized and the size of the coefficients is reduced. Following Forte et al. (2017), the cross-correlation was computed both for the F0 band-passed audio feature and its Hilbert transform, and the terms were interpreted as the real and imaginary part of a complex cross-correlation function. The magnitude of this complex function was then interpreted as the neural response.

For completeness, we also computed the cross-correlation for the broadband feature, as well as the regression model for the F0 feature. As features may interact with model regularization (as further discussed below) we investigated regression models both without and with high degrees of regularization.

### 2.4. Response Peak Statistics

Response peaks for the subcortical analyses were extracted between 5 and 11 ms, and cortical envelope and relative pitch analyses in ranges 100–210 and 50–160 ms, respectively. For all subcortical analyses, activity measured at the vertex electrode Cz was analyzed, to mimic clinical ABR recording settings. For the cortical approaches, the analysis was performed on the average over six auditory-relevant frontal electrodes (FC1, FC2, FC5, FC6, F3, F4; Di Liberto et al., 2015; Hjortkjær et al., 2018). For each participant, the maximum of the response in the pre-defined time window was interpreted as the response peak. One-tailed Pearson correlations between peak amplitudes and latencies within participants were calculated. All presented p-values were corrected for multiple comparisons according to false discovery rate (FDR) following Benjamini and Yekutieli (2001).

### 2.5. Model Comparisons

A critical question was whether a unique contribution of the different pitch and envelope-related features to the EEG response can be separated. The F0 band-passed and broadband audio features may be mutually correlated, making it difficult to associate responses to unique variance. Following previous studies (Di Liberto et al., 2015; Teoh et al., 2019), we investigated this by combining features and computing the improvement in prediction performance of the combined models relative to models containing the individual features separately. If the combined model significantly outperforms the individual feature models, then both features may provide a unique contribution to the prediction. For the subcortical features, we thus regressed the broadband EEG on the broadband rectified speech waveform, the F0 band-passed speech signal, or the two features combined. To accommodate for the different regularization parameters associated with the features, we fitted the regularization parameter λ similar as described above for the cortical analysis, but on time lags between 2.44 and 12.21 ms. In the cortical analysis, we similarly combined the low-pass filtered envelope and the relative pitch feature to investigate their relative contribution (cf. Teoh et al., 2019).

### 2.6. Simulations

Model accuracy, however, depends on the SNR of the measured neural response. To investigate the degree to which the unique contribution of the considered features can be partialed out over different SNRs, we further performed model simulations. Data simulations were performed by adding noise with a 1/f distribution (EEG-shaped) at various SNRs to the speech features, convolved with a predetermined TRF, and then computing the regression analysis as described above. As results remained unchanged when fitting regularization independently for different regressors, regularization was fitted jointly within combined models. Any increase in prediction performance of the combined models were computed relative to the individual feature models, as in the EEG data analysis. We also compared individual feature models with combined models where a feature was combined with a random Gaussian signal in the simulated data. This allowed us to estimate the upper bound of model improvement with uncorrelated features for a given SNR.

## 3. Results

### 3.1. Subcortical Responses

**Figure 3:**
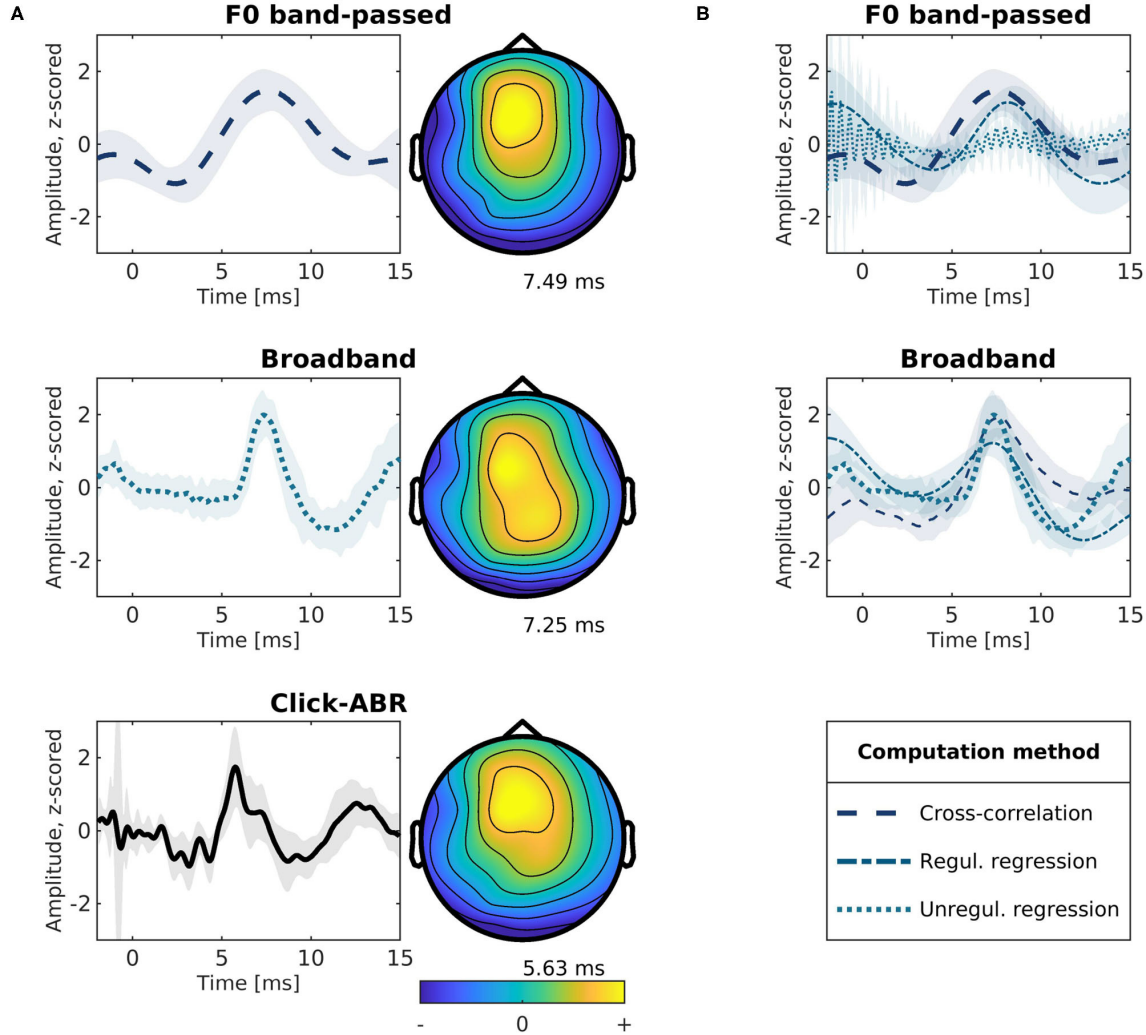
Modeled subcortical responses to different speech features. All traces show the response at electrode Cz. Shaded areas indicate ±1 standard error of the mean across participants. **(A)** Modeled responses for different speech features and topographies at mean peak latencies. Top: Complex cross-correlation between the F0 band-passed speech and the EEG (80–240 Hz). Middle: The EEG (>80 Hz) regressed onto the broadband (>80 Hz) rectified speech signal using ordinary least-squares regression. Bottom: Conventional click-evoked ABRs. **(B)** Subcortical response functions modeled using the F0 band-passed (top) or broadband features (middle) and estimated using cross-correlation or regression. For regression, both the unregularized ordinary least-squares and a highly regularized solution (λ = 10^9^) is shown.

Subcortical responses obtained with the different speech features and their topographies are presented in Figure 3A. We first examined responses found by linearly regressing the running EEG on the rectified broadband waveform of the running speech signal (similar to Maddox and Lee 2018). TRFs for this broadband signal (middle left panel) show a distinct wave-V-like peak at 6.76–8.47 ms in latency. We compared these speech-derived TRFs to standard click-evoked ABRs (Figure 3A bottom left). Within individual subjects, the response peaks identified with the broadband speech signal correlated with wave V in click-evoked ABRs (5.38–6.36 ms) in terms of latency (*p* = 0.039, ρ = 0.598), and amplitude (*p* < 0.001, ρ = 0.706). The complex cross-correlation between the running EEG with the F0 band-passed running speech signal (similar to Forte et al., 2017) is shown in the top left panel in Figure 3A. The magnitude of the complex cross-correlation function showed a peak at 5.05–9.44 ms in latency. Peaks in this range were also correlated with wave V of the click-ABRs within subjects in terms of amplitude (*p* < 0.001, ρ = 0.760), but not latency (*p* > 0.05). Similarly, responses to the broadband and the F0 band-passed speech were mutually correlated in amplitude (*p* < 0.001, ρ = 0.744), but not in latency (*p* > 0.05). For comparison, Figure 3B shows responses to each of the two speech features (F0 band-passed and the broadband signal) estimated with either regression or the complex cross-correlation function. For regression, both the unregularized, ordinary least squares and a highly regularized solution is shown.

### 3.2. Distinct Tracking of F0?

While responses extracted by regressing the EEG onto the rectified broadband speech waveform were consistent with ABR wave V (Figure 3 mid and bottom), as previously reported (Maddox and Lee, 2018), it remains unclear whether subcortical tracking of the F0 periodicity from the running speech signal can be extracted separately. As can be seen in Figure 3A (top left), the cross-correlation of the F0 band-passed audio with the EEG response suggests a more smooth waveform with later response peaks, as also reported in previous work (Forte et al., 2017; Etard et al., 2019; Van Canneyt et al., 2021a,b,c). To investigate their unique predictive power, we linearly combined the broadband and F0 band-passed signals in a regression model to jointly predict the subcortical EEG response. We then tested for improvement in prediction accuracy relative to the individual models (following e.g., Di Liberto et al., 2015; Teoh et al., 2019). The rectified broadband signal predicted the EEG significantly better than the F0 band-passed speech (*p* = 0.008). Yet, no significant improvement was found by combining the features (*p* > 0.05). Thus, adding F0 to a model of the broadband rectified waveform yielded no additional predictive power.

**Figure 4:**
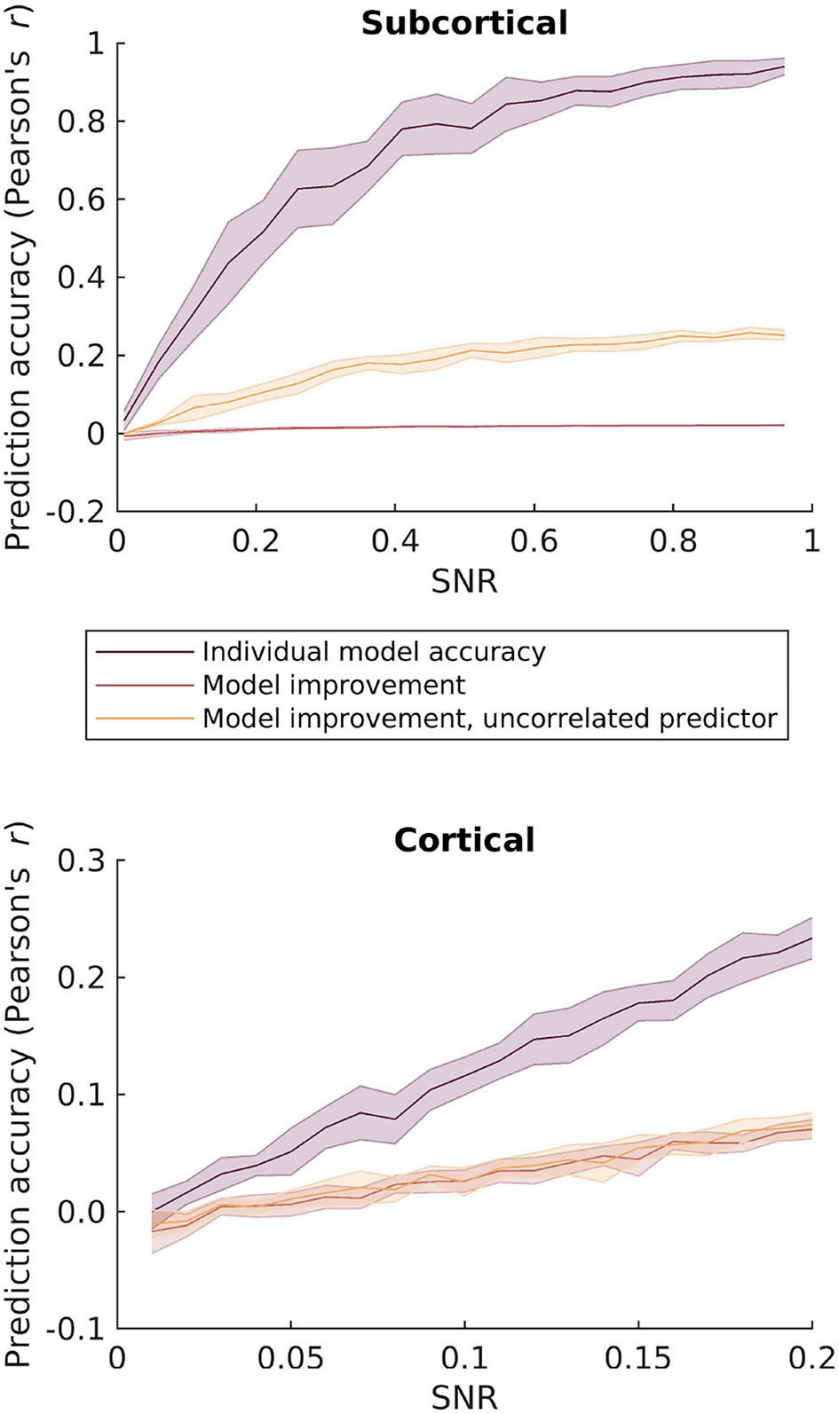
Simulated prediction accuracies for the different envelope and pitch-related features as a function of SNR. Top: comparison of the F0 band-passed and broadband rectified speech features considered in the subcortical response analysis. Bottom: comparison of the envelope and relative pitch features considered in the cortical analysis. Purple: Simulated regression prediction accurracies for the best performing individual feature model. Red: Improvement in prediction accuracy by combining the two features relative to the best performing individual model. Yellow: Improvement in prediction accuracy by combining the relevant features with an uncorrelated predictor as an estimate of the upper limit of prediction improvement.

This is likely due to the fact that the two speech features are mutually highly correlated. To investigate this further, we simulated responses to the two speech features by adding EEG-shaped noise to the features. We then computed model improvement by comparing the combined broadband and F0 features relative to the individual models as a function of SNR. As can be seen in the top panel of Figure 4, even at high SNRs the combined model leads to almost no improvement in prediction accuracy compared to the individual feature models. This again suggests that dissociating subcortical F0-tracking responses may be challenging with running speech.

**Figure 5:**
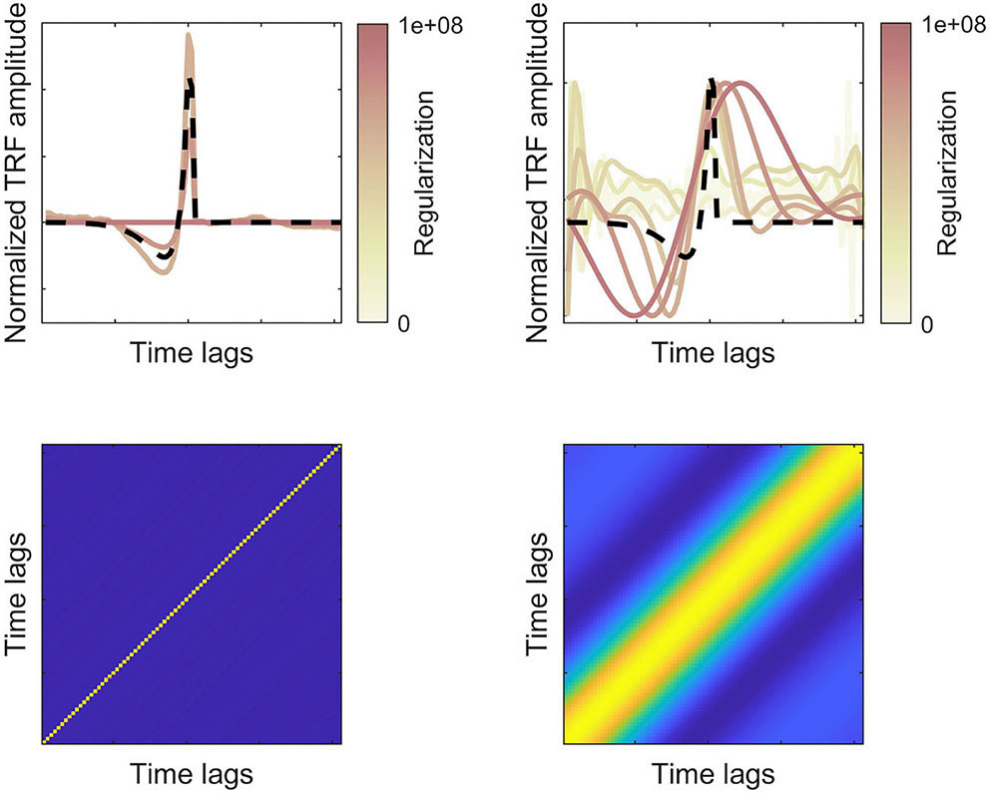
Simulated TRF responses for features with lower (left) or higher (right) degrees of autocorrelation. Bottom panels show the autocorrelation matrices of two simulated features (filtered random Gaussian variables). Top panels show the true (dashed lines) and estimated TRFs for different degrees of regularization (normalized amplitudes). For the more autocorelated feature, higher regularization is required to estimate the true TRF, but overregularization leads to temporal smearing of the response function.

Given that the F0 band-passed and broadband rectified speech waveforms are highly correlated, it is perhaps surprising to find that the F0-responses (Figure 3A, top left) yield later peaks and more smooth response. However, these differences may stem from differences in the autocorrelation of the features and how the autocorrelation is dealt with in the stimulus-response model (Crosse et al., 2016). The low-pass filter around F0 effectively enhances signal autocorrelation, compared to the broadband speech signal, which must be compensated for in the stimulus-response model. Following Forte et al. (2017), the F0-response was estimated using cross-correlation, while the broadband feature was estimated with regression (Maddox and Lee, 2018). In the framework of regularized regression (Equation 1), this corresponds to different amounts of regularization (λ) controlling the autocorrelation term in the regression. Figure 3B showing both the regularized and unregularized solutions suggested that the F0 band-passed feature requires a higher degree of regularization. The effect of regularization for features with different degrees of autocorrelation is illustrated with simulated data in Figure 5. Here, we simulate how well a true TRF peak (dashed lines) can be estimated given different degrees of feature autocorrelation. Compared to a broadband “white” feature (left panels), a low-passed signal with higher autocorrelation (right panels) requires regularization to estimate the true TRF peak latency. However, very high degrees of regularization (corresponding to cross-correlation) with an autocorrelated regressor smears the estimated response function and may consequently shift the response peaks (Figure 5, top right). Higher degrees of autocorrelation in the F0 band-passed signal in combination with cross-correlation may thus potentially explain the observed differences in peak latencies.

### 3.3. Cortical Pitch Processing

**Figure 6:**
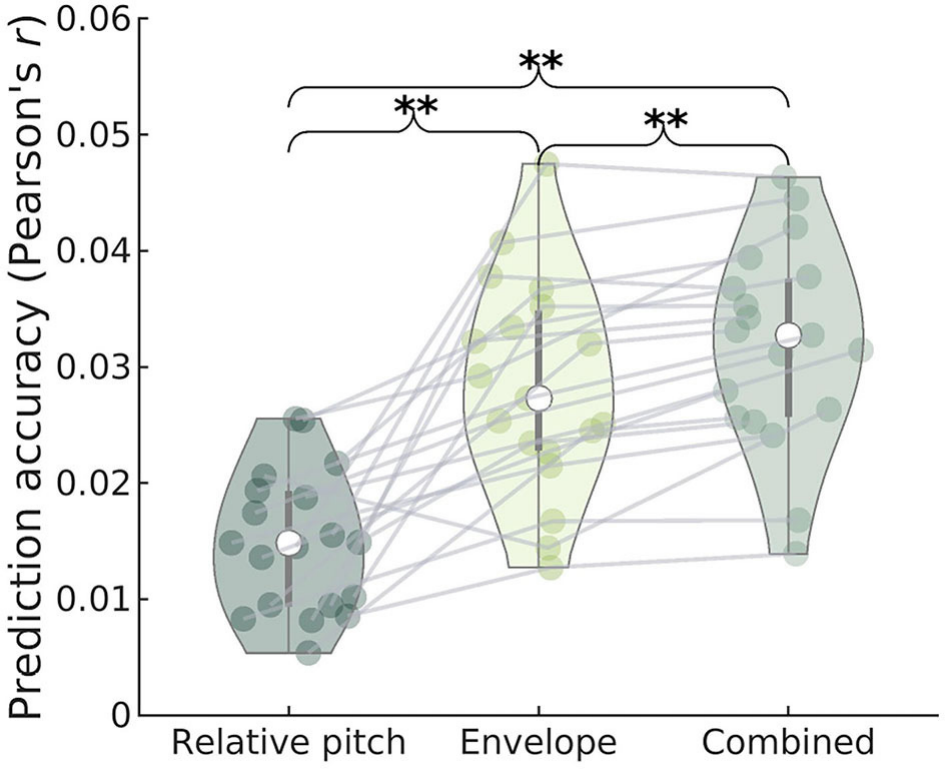
Prediction accuracy (Pearson's *r*) of cortical EEG for regression models containing relative pitch, envelope, or both features as predictors. Gray lines indicate individual subject data. The combined model yields significantly higher accuracy compared to either of the individual models, suggesting that both predictors explain a unique part of the variance. The envelope model shows significantly higher prediction accuracy compared to the relative pitch model.

We also investigated whether cortical activity tracks the relative pitch contour of the running speech signal, as proposed in recent work (Tang et al., 2017; Teoh et al., 2019). As for the subcortical features, we investigated the degree to which relative pitch predicts unique variance in the cortical EEG after accounting for envelope tracking. We therefore regressed the cortical low-frequency EEG separately on relative pitch and the low-frequency envelope, as well as the two features combined. As seen in Figure 6, we found that adding relative pitch to an envelope model led to a significant improvement in prediction (paired permutation test, all *p* = 0.005), replicating the findings of Teoh et al. (2019).

Unlike the subcortical features, this indicates that the relative pitch track is sufficiently uncorrelated with envelope fluctuations for allowing these two features to be dissociated in the cortical response. Again, we simulated the effect of combining envelope and pitch features over a range of SNRs. As can be seen in the bottom panel in Figure 4, combining the two cortical features leads to an increase in model accuracy as SNR increases. Importantly, this improvement with increasing SNR was similar when simulating a random feature that is uncorrelated with the speech features. The model improvement is significantly larger than zero already at low SNRs, where the prediction accuracy corresponds to that found with real EEG data.

Reasoning that tracking of the periodicity at F0 in the brainstem may provide a temporal code for relative pitch-tracking in cortex, we tested whether subcortical response peaks to F0 band-passed speech correlated with the cortical response to relative pitch. Across participants, responses to relative pitch yielded a positive TRF peak at frontal electrodes around 100 ms latency (77.07–139.57 ms), consistent with Teoh et al. (2019). No correlation was found between this cortical response peak and the subcortical response peak to the F0 band-passed speech determined with the complex cross-correlation function (Figure 3A, top panel), neither in terms of latency nor amplitude (*p* > 0.05). Similarly, we tested whether the ABR V-like peaks of the subcortical responses modeled to rectified broadband speech (Figure 3A, middle panel) were correlated with cortical responses to the envelope. Cortical responses to the envelope showed a positive peak around 140 ms latency (123.95–186.45 ms) at frontal electrodes, consistent with previous work (e.g., Fuglsang et al., 2017). No significant correlation in peak latency or amplitude was found (both *p* > 0.05) for this comparison either.

## 4. Discussion

Our results replicate those of Maddox and Lee (2018) showing that regressing subcortical EEG responses onto the rectified running speech waveform yields response functions consistent with the ABR wave V and correlated with conventional click-ABRs. Measuring unique F0-related subcortical responses may be challenging. In the cortex, on the other hand, our results also replicated previous work suggesting distinct processing of relative pitch (Teoh et al., 2019).

The approaches evaluated here propose objective measures of subcortical running speech processing, which may offer new research possibilities and potentials for clinical applications. Brainstem EEG is used for objective hearing assessment in patient groups where the response-reliant pure-tone audiometry might not be applicable. For example, brainstem measures are used for hearing screening in newborns (World Health Organization, 2010; Patel et al., 2011). The use of continuous speech instead of clicks or tone beeps may become relevant for patient groups in need of a more engaging assessment design to uphold compliance (e.g., children), or patients that may be uncomfortable with unfamiliar sounds (e.g., patients with dementia). Furthermore, subcortical measures of running speech can potentially serve as an objective tool to evaluate and tailor hearing assistive devices (HADs) to the user's needs. Signal processing in hearing aids is typically programmed to process and enhance real-world speech, and brainstem responses may thus be obtained with the stimulus they are designed for. This is especially relevant for the evaluation of hearing aid noise suppression schemes that might classify non-speech or monotonously repeating stimuli as noise.

Evoked responses from the auditory brainstem can be measured both with broadband transient stimuli, as in click-ABRs, and with periodic stimuli, as in tone- or speech-evoked FFRs. The degree to which ABRs and FFRs rely on the same generators is debated. Wave V of the ABR is most efficiently elicited by broadband signals like clicks or chirps (Dau et al., 2000), and high-frequency stimulation contributes significantly to the response magnitude of wave V (Wegner and Dau, 2002). FFRs typically require relatively high sound pressure levels to be elicited and are usually not observed near threshold (Krishnan and Parkinson, 2000; Bidelman and Powers, 2018), which may indicate that the FFR to low-frequency tones or speech F0 is not a direct neural correlate of on-frequency processing, but requires synchronous activity from mid- and high-frequency peripheral neural channels (Wegner and Dau, 2002; Dau, 2003). Dau (2003) showed that a unitary response obtained by deconvolution of click-ABR data with simulated auditory nerve activity from a level-dependent and frequency-selective model can predict both click-ABRs and tone FFRs. This indicates that the frequency-following activity measured with EEG may not reflect pitch-specific or on-frequency processing, but rather reflects summed neural activity across cochlear frequency channels. These conclusions are consistent with the current study indicating that F0 yields no separate predictive power for the subcortical response compared to the broadband speech signal. Instead, we find that including high-frequency information via the broadband signal improves prediction of the subcortical response relative to prediction with the F0. The cortical response analysis, on the other hand, replicated the findings of Teoh et al. (2019) indicating distinct processing of envelope and pitch features. The combined model containing both relative pitch and the low-pass filtered speech envelope as predictors significantly outperformed both individual models for cortical features, which could be supporting evidence of distinct processing of relative pitch in the cortex. However, we can not rule out the possibility that the relative pitch feature is correlated with other features that the models do not account for. Simulations indicated that the model improvement by combining relative pitch and the low-passed amplitude envelope is comparable to the effect of adding an uncorrelated feature. The dissociation of envelope and pitch processing is a potentially attractive tool for research on tonal languages, where the F0 track not only conveys prosodic, but also lexical information. For example, it offers perspectives for objectively measuring the processing of lexical tone in listeners with elevated hearing thresholds.

## Data Availability Statement

The datasets presented in this article are not readily available because written consent from the participants to share data was not obtained. Requests to access the datasets should be directed to Florine L. Bachmann, flbach@dtu.dk.

## Ethics Statement

The studies involving human participants were reviewed and approved by Science-Ethics Committee for the Capital Region of Denmark (reference H-16036391). The participants provided their written informed consent to participate in this study.

## Author Contributions

FB collected and analyzed the data and drafted the manuscript. JH performed simulations. All authors contributed to the article and approved the submitted manuscript.

## Funding

This work was partially funded by Sonova AG (FB) and by the Novo Nordisk Foundation Synergy grant NNF17OC0027872 (UHeal) (JH).

## Conflict of Interest

The authors declare that this study received funding from Sonova, a major hearing care solutions company (FB). The funder was not involved in the study design, collection, analysis, interpretation of data, the writing of this article or the decision to submit it for publication. The authors declare that the research was conducted in the absence of other commercial or financial relationships that could be construed as a potential conflict of interest.

## Publisher's Note

All claims expressed in this article are solely those of the authors and do not necessarily represent those of their affiliated organizations, or those of the publisher, the editors and the reviewers. Any product that may be evaluated in this article, or claim that may be made by its manufacturer, is not guaranteed or endorsed by the publisher.
